# Alternatively spliced *ELAVL3* cryptic exon 4a causes ELAVL3 downregulation in ALS TDP-43 proteinopathy

**DOI:** 10.1007/s00401-024-02732-y

**Published:** 2024-05-30

**Authors:** Isabel Costantino, Alex Meng, John Ravits

**Affiliations:** 1 Department of Neurosciences, ALS Translational Research, University of California San Diego, La Jolla, CA USA; 2 Neurosciences Graduate Program, University of California San Diego, La Jolla, CA USA; 3 Medical Scientist Training Program, School of Medicine, University of California San Diego, La Jolla, CA USA

Amyotrophic lateral sclerosis (ALS) is a progressive neurodegenerative disease characterized clinically by progressive weakness and neuropathologically by nuclear loss and cytoplasmic aggregation of the RNA binding protein TDP-43 in neurons of the brain and spinal cord in > 97% of cases [6]. TDP-43 is mainly a nuclear protein and plays a major role in RNA splicing. Loss of nuclear functions may lead to splice defects, such as insertion of noncanonical sequences called cryptic exons (CEs) that may cause premature stops, polyadenylation, truncation or non-functional proteins [4]. Two of the most recently identified and best characterized examples of cryptic exon inclusion are *STMN2* CE 2a, leading to premature polyadenylation and a premature stop sequence, and *UNC13A* CE 21a, leading to a premature stop sequence [1, 3, 5, 7]. We previously reported downregulation and nuclear loss of the RNA binding protein *ELAVL3* at both the transcript and protein levels in ALS nervous systems [2]. Loss of ELAVL3 in mice results in axonal deformity, loss of neuronal polarity, and synapse formation deficits [8]. Here we use reverse transcription quantitative PCR (qRT-PCR) and chromogenic in situ hybridization (CISH) applied to the spinal cord and motor cortex and show that the mechanism involved in this downregulation is also related to expression of a CE.

Within intron 3 (between exons 3 and 4) of *ELAVL3* there is an intronic binding domain for TDP-43 (Fig. 1a). Expression of a cryptic exon upstream of the TDP-43 binding site, *ELAVL3* CE 4a, is seen when TDP-43 is reduced in a TDP-43 knock-down cellular model and in TDP-43 negative neuronal nuclei from frontotemporal lobar degeneration frontal cortex (Fig. 1b) [3, 10]. Using RT-PCR and Sanger sequencing, we confirmed the existence of a 166-nucleotide sequence in mRNA transcripts, *ELAVL3* CE 4a (hg38 chr19:11,463,662–11,463,496) in ALS tissues and note that this creates a frameshift leading to multiple premature stop sequences (Fig. 1c). We re-confirmed our previous data demonstrating downregulation of total *ELAVL3* in ALS spinal cord, and we expanded these findings into motor cortex to show reduced *ELAVL3* mRNA in ALS relative to controls (Fig. 1d). We detected *ELAVL3* CE 4a using a short segment containing CE 4a and exon 4 by qPCR in 43% of ALS spinal cord and 77% of ALS motor cortex samples (Fig. 1e, Table 1). Expression of *ELAVL3* transcripts containing CE 4a was extremely low relative to total *ELAVL3*. We also detected *ELAVL3* CE 4a in 13% (2/15) spinal control cases, perhaps related to age (82 and 76 years compared to control mean of 62 years).

**Fig. 1:**
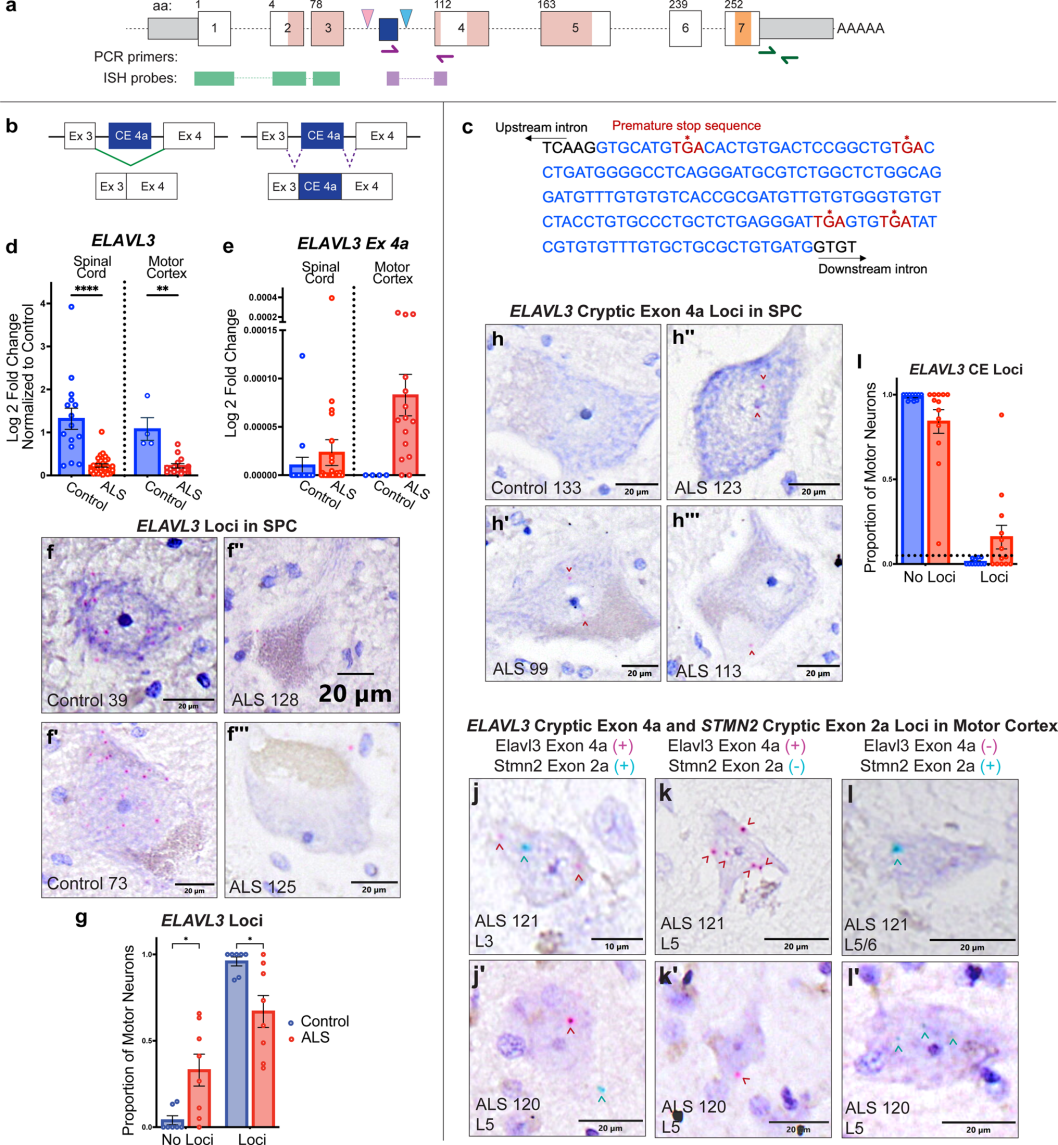
Cryptic exon 4a in *ELAVL3* mRNA is alternatively spliced and expressed in ALS tissue. (**a**) *ELAVL3* (Hg38, chr19:11,449,326–11,483,046) contains two RNA binding domains (pink), one RNA binding domain with poly(A) binding domains (orange), a cryptic exon between canonical exons 3 and 4 (“CE 4a”) (dark blue), and binding domains for TDP-43 (light blue triangle) and neuronal ELAVL proteins (nELAVL, pink triangle). Quantitative PCR primers detecting total *ELAVL3* mRNA (green) and *ELAVL3* containing cryptic exon 4a (purple) are shown as half-arrows. In situ hybridization probes detecting total *ELAVL3* mRNA (targeting a region containing the 5′UTR and exons 1–3) (green) and *ELAVL3* containing cryptic exon 4a (targeting the junction between cryptic exon 4a and exon 4) (purple) are shown as rectangular segments. (**b**) Normal splicing of *ELAVL3* involves canonical exons 3 and 4 (left); in ALS, there is abnormal splicing of the cryptic exon in intron 3 between them (right). (**c**) Sanger sequencing of *ELAVL3* cryptic exon 4a (blue) reveals several premature stop sites (red letters with asterisks). (**d**) qPCR demonstrates higher expression of total *ELAVL3* mRNA in controls compared to ALS in spinal cord (*P* < 0.0001) and motor cortex (*P* = 0.008). (**e**) qPCR demonstrates *ELAVL3* cryptic exon 4a expression at low levels in ALS spinal cord and motor cortex. (**f**) Chromogenic in situ hybridization shows total *ELAVL3* loci in both control (**f**–**f**′) and ALS (**f**″–**f**‴) anterior horn lower motor neurons (total *ELAVL3* signal is red, and counterstain is purple). (**g**) The proportion of motor neurons with detectable total *ELAVL3* loci by chromogenic in situ hybridization is significantly lower in ALS (mean = 0.67) than controls (mean = 0.96) (*P* = 0.04). (**h**) Chromogenic in situ hybridization using probes to *ELAVL3* cryptic exon 4a detects alternatively spliced *ELAVL3* containing cryptic exon 4a in control (**h**) and ALS (**h**′-**h**‴) spinal cord anterior horn motor neurons (signal is red and highlighted with a red arrowhead and counterstain is purple). (**i**) ALS has a higher proportion of motor neurons positive for *ELAVL3 c*ryptic exon 4a than controls (*P* = 0.1). The threshold of detection is 0.05 in this assay (shown in dotted line)–data points above threshold are considered positive for *ELAVL3* cryptic exon 4a. (**j**–**l**′) Duplex chromogenic in situ hybridization detects *ELAVL3* CE 4a (red) and STMN2 CE 2a (blue) in the motor cortex including neurons positive for both CEs (**j**–**j**′), positive only for *ELAVL3* CE 4a (**k**–**k**′), and positive only for STMN2 CE 2a (**l**–**l**′). The cortical layer is indicated in bottom left. All graphs plotted as mean ± SEM

**Table 1:** *ELAVL3* cryptic exon 4a expression

	RT-PCR		In situ* hybridization*	
Diagnosis	Spinal cord	Motor cortex	Spinal cord	Motor cortex
ALS	13/30 (43.4%)	7/9 (77.8%)	6/13 (46.2%)	4/6 (66.7%)
Control	2/15 (13.3%)	0/5 (0%)	0/11 (0%)	0/3 (0%)

In order to visualize cell specificity of *ELAVL3* expression, we performed CISH in formalin-fixed, paraffin embedded tissue sections using a BaseScope™ assay. We selected regions in ALS cases with preserved numbers of motor neurons, reasoning that early molecular events at the neuronal level were more likely to be identified in regions of the nervous system with the highest numbers of residual motor neurons [9]. For total *ELAVL3* mRNA, we used a probe targeting a 300 base-pair region containing exons 1, 2, and 3. In ALS spinal cord, there were significantly fewer motor neurons positive for total *ELAVL3* loci compared to control–68% of motor neurons in control contain 4 or more loci compared to 41% in ALS (Fig. 1f–g). This is consistent with *ELAVL3* mRNA downregulation in ALS.

To visualize cell specificity of *ELAVL3* CE 4a expression by CISH, we used a probe that targets a 50-nucleotide RNA sequence containing the junction of exon 4a and exon 4 (Fig. 1a). We found 46% of ALS spinal cord and 67% of motor cortex cases were positive for expression of *ELAVL3* exon 4a loci (Fig. 1h–i, Supplementary Fig. 1a–d′, Table 1). The majority of ALS motor neurons expressing ELAVL3 CE 4a contained between 1–3 loci (mean: 15.4% of total spinal cord motor neurons). In the spinal cord, *ELAVL3* CE 4a were seen in anterior horn motor neurons and smaller neurons in the posterior regions of the anterior horn. We did not detect *ELAVL3* exon 4a loci in glia of white matter tracts or in posterior horn neurons. In the motor cortex, we rarely detected expression of *ELAVL3* CE 4a in motor neurons (Betz cells). Rather, we detected expression predominantly in smaller neurons of layers 3, 5, and 6 (Fig. 1j–k). We did not detect *ELAVL3* CE 4a loci above assay threshold in control nervous systems.

For comparison, we measured *STMN2* CE 2a expression in our cohort by qPCR and CISH. By qPCR, full-length *STMN2* was reduced in both ALS spinal cord and motor cortex, and *STMN2* CE 2a was detected in 93% of ALS spinal cords and motor cortices (Supplementary Fig. 2a–b). Similarly, using a CISH probe targeting 300 nucleotides of the 3′ untranslated region and a probe targeting a 50-nucleotide sequence containing the junction of canonical exon 1 and CE 2a, respectively, we detected downregulation of *STMN2* and expression of *STMN2* CE 2a in spinal cord motor neurons (Supplementary Fig. 2c–d). Approximately a quarter of spinal cord motor neurons contained *STMN2* exon 2a (Supplementary Fig. 2e–f). In the motor cortex, we detected a pattern of expression of *STMN2* CE 2a similar to *ELAVL3* CE 4a. We did not detect *STMN2* CE 2a in control nervous systems.

Using a duplex CISH assay to detect *ELAVL3* CE 4a and *STMN2* CE 2a simultaneously, there was not a strong concordance between expression of the CEs (Fig. 1j–l, Supplementary Fig. 1e–i). This suggests that neuronal subtypes have desynchronization of molecular changes, although we are at the technical limits to detect differential sensitivity of TDP-43 disruptions. The higher expression of transcripts containing *STMN2* CE 2a relative to those containing *ELAVL3* CE 4a fits with the pattern of the hypothesized destinations of these transcripts. *STMN2* is a highly expressed transcript in anterior horn spinal cord, and the inclusion of the CE causes premature polyadenylation and inclusion of a premature stop sequence. *ELAVL3* is moderately expressed relative to *STMN2* and inclusion of *ELAVL3* exon 4a leads to premature stop codons.

In summary, we confirm there is a CE in intron 3 of *ELAVL3* that is expressed in ALS spinal cord and motor cortex using multiple modalities (Table 1). We consider the overall reduction of *ELAVL3* expression is by way of this splice dysregulation and CE expression, generating mRNA with premature stop sequences, similar to what has been reported for *UNC13A* and *STMN2*. Interestingly, although we presume the *ELAVL3* CE expression is related to loss of TDP-43, we cannot entirely exclude the possibility that expression reduction relates to the dysfunction of ELAVL protein itself, since ELAVL2, ELAVL3, and ELAVL4 also bind intron 4 and thus could modulate its own splicing (Fig. 1a).

### Supplementary Information

Below is the link to the electronic supplementary material.

Supplementary file1 (DOCX 4593 KB)

Supplementary file 2 (XLSX 20 KB)

## Data Availability

Images are available upon request.
