# Supplementary material for: Alternatively spliced ELAVL3 cryptic exon 4a causes ELAVL3 downregulation in ALS TDP-43 proteinopathy
Source: Acta Neuropathol. 2024 May 30;147(1):93. doi: 10.1007/s00401-024-02732-y (PMC11139733; doi:10.1007/s00401-024-02732-y)
Supplement: Supplementary file 1 — Supplementary file1 (DOCX 4593 KB) [file 401_2024_2732_MOESM1_ESM.docx]

**
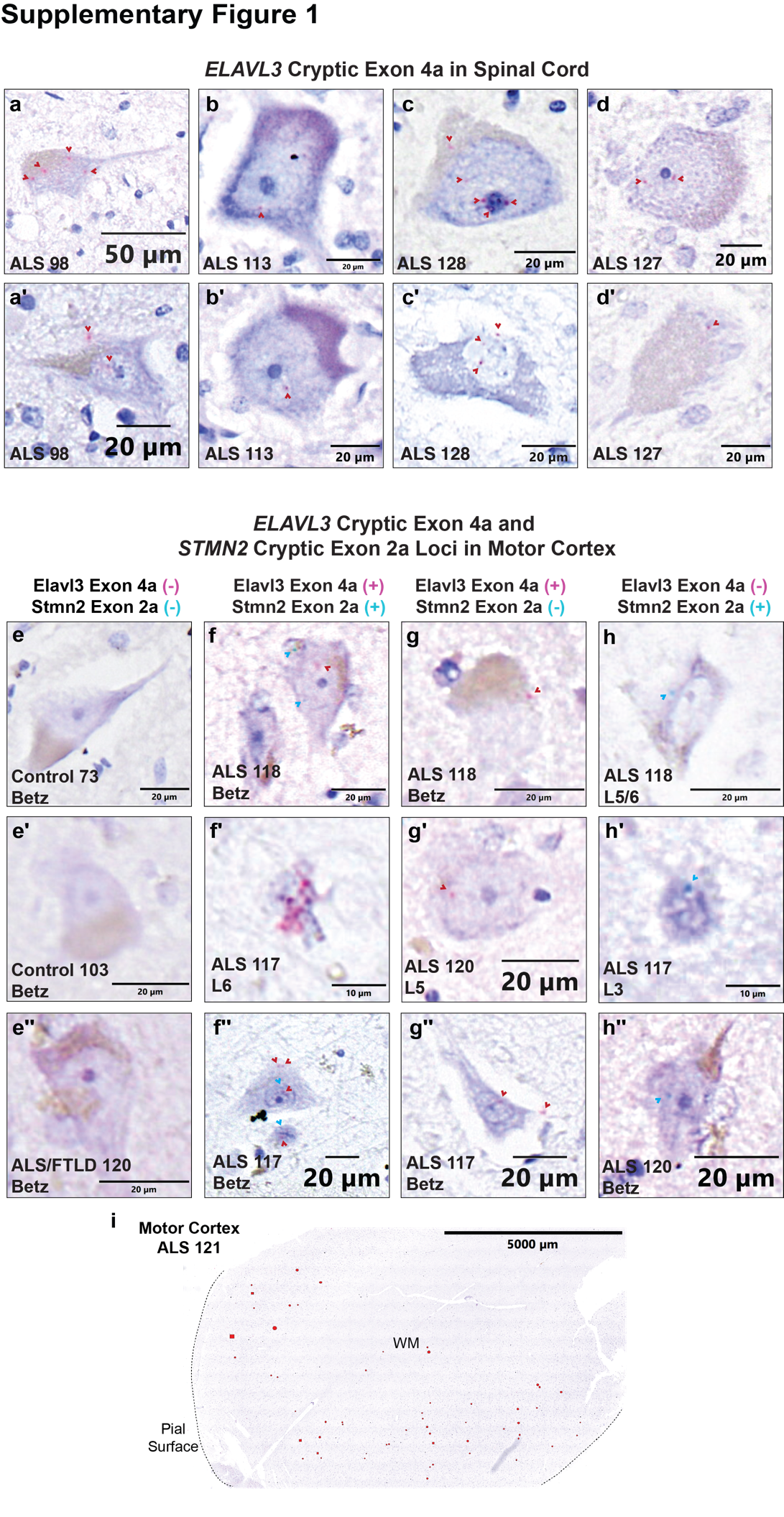
**

**Supplementary Fig 1 Additional representative images of *ELAVL3* cryptic exon 4a and *STMN2* cryptic exon 2a expression by *in situ* hybridization**

**(a-d')** Additional representative images of *ELAVL3* CE 4a expression in anterior horn motor neurons of ALS spinal cord using *in situ* hybridization using a single channel chromogenic BaseScope assay. Signal in red, counterstain in purple. **(d-h''')** Additional representative images of *ELAVL3* CE 4a (red) and/or *STMN2* CE 2a (blue) expression in ALS motor cortex using a duplex chromogenic BaseScope assay. Counterstain in purple. **(i)** Representative snapshot of the frequency of *ELAVL3* CE 4a and/or *STMN2* CE 2a expression events in ALS motor cortex. Red-filled shapes drawn over cells for visualization.

**
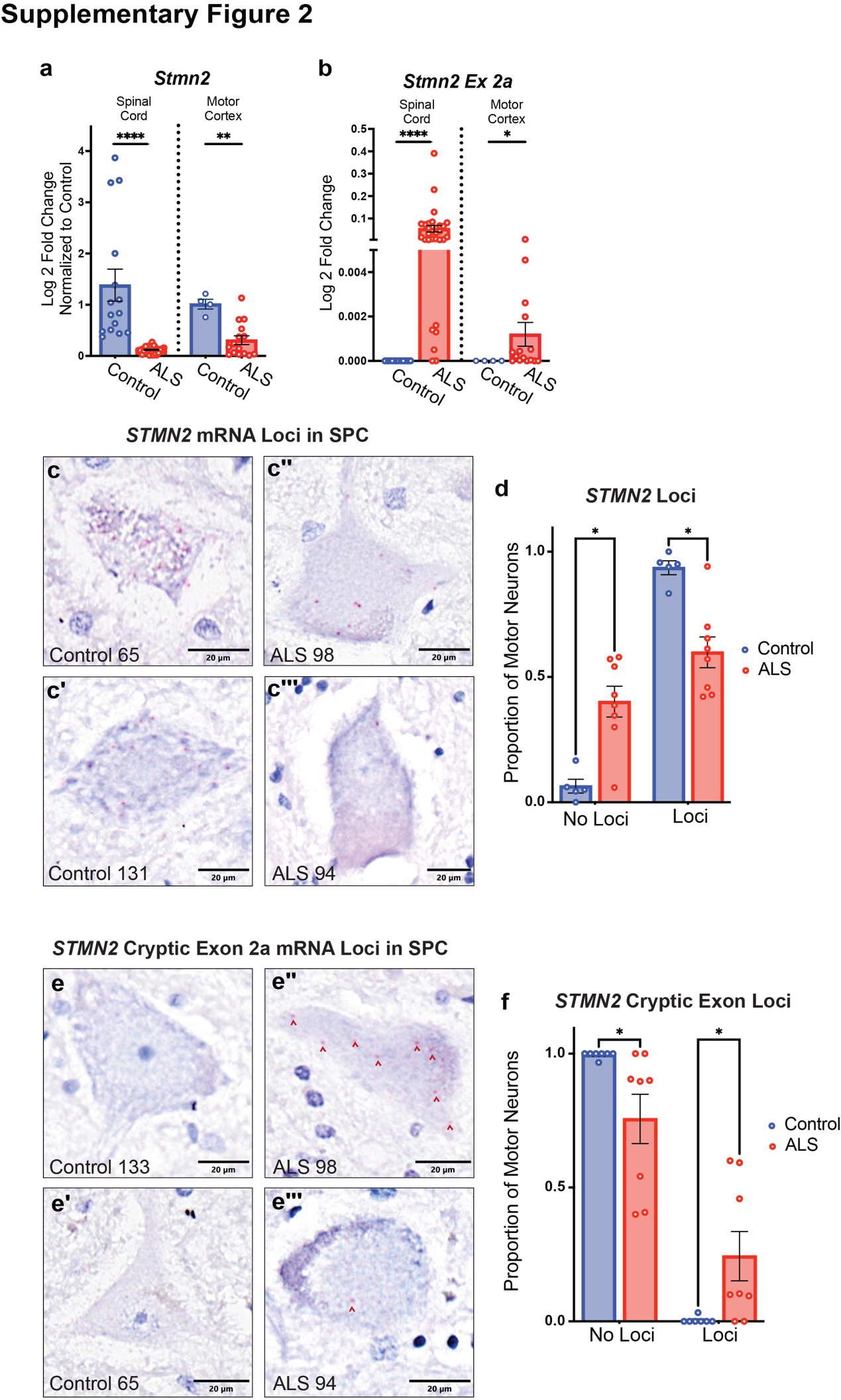
**

**Supplementary Fig 2 *STMN2* mRNA is reduced and *STMN2* cryptic exon 2a is expressed in spinal cord in ALS**

**(a)** qPCR in spinal cord and motor cortex using primers amplifying a small segment of *STMN2* 3**´**UTR. Control spinal cord and motor cortex showed higher expression of *STMN2* compared to ALS (P<0.0001, P=0.004). **(b)** qPCR in spinal cord and motor cortex using primers amplifying a small segment from *STMN2* Exon 1 to Exon 2a shows no expression in the controls and significantly more expression in spinal cord and motor cortex (P<0.0001, P=0.01). **(c-d)** Chromogenic *in situ* hybridization (CISH) captures expression reduction of *STMN2* mRNA and expression of *STMN2* cryptic exon 2a in ALS spinal cord motor neurons. Signal in red, counterstain in purple. (**c-c'''**) Representative images of *STMN2* 3**´**UTR mRNA in control and ALS spinal cord (**d**) The proportion of motor neurons with measured *STMN2* loci was significantly higher in controls compared to ALS (mean = 0.9) compared to ALS (mean=0.60, *P=0.0124). **(e-f)** CISH probing for a segment of *STMN2* mRNA containing the junction of exon 1 and cryptic exon 2a in control and ALS spinal cord. Signal in red, counterstain in purple. (**e-e''')** Representative images of *STMN2* exon 2a loci in control (**e-e'**) and ALS (**e''-e'''**) motor neurons. **(f)** ALS spinal cord had significantly more motor neurons positive for cryptic exon 2a (mean=0.2437) compared to controls (mean=0.005, *P=0.02). All graphs plotted as mean +/- SEM.
